# Assessing the solar variability signature in climate variables by information theory and wavelet coherence

**DOI:** 10.1038/s41598-021-90044-6

**Published:** 2021-05-31

**Authors:** Ileana Mares, Venera Dobrica, Constantin Mares, Crisan Demetrescu

**Affiliations:** Institute of Geodynamics, Romanian Academy, Bucharest, Romania

**Keywords:** Climate sciences, Hydrology

## Abstract

The present study aims to investigate the possible influence of solar/geomagnetic forcing on climate variables, such as the drought index, Danube discharge and large-scale atmospheric indices. Our analysis was performed separately for each season for two time periods, 1901–2000 and 1948–2000. The relationship between terrestrial variables and external indices was established based on the application of (1) information theory elements, namely, synergy, redundancy, total correlation, transfer entropy and (2) wavelet coherence analysis. Bandpass filtering has also been applied. The most significant signature of the solar/geomagnetic forcing in the climate variables was obtained for the data smoothed by the bandpass filter. According to our results, significant solar/geomagnetic forcing appears in the terrestrial variables with a delay of 2–3 years.

## Introduction

Although great efforts have been made to identify a robust link between solar/geomagnetic activity and terrestrial variables, the results are controversial because inadequate statistics and inappropriate procedures were applied. Several studies^1–12^ have taken into account the signature of solar activity according to its manifestations on terrestrial variables. However, Lockwood (2012)^8^ critically points out that if the increase in temperature due to solar activity is difficult to determine on a global scale, then such work can be performed on a regional scale by neglecting certain factors, such as greenhouse effects, and explaining the physical mechanisms; for example, winter temperatures in the Eurasian area are influenced by solar activity. The physical mechanisms that favour the impact of stronger solar activity in the Eurasian area relative to other regions are explained by several authors^5,7,13,14^. These mechanisms mainly refer to the high solar-atmosphere interaction through the so-called “top-down” mechanism, whereby stratospheric changes influence the underlying troposphere, and the influences of thermal advection and land-sea thermal contrast are added. In Barriopedro et al. (2008)^15^ it is shown the modulatory role that the Quasi-Biennial Oscillation (QBO) has on the solar influence in the occurrence of atmospheric blocking events.

Due to the multiple approximations that need to be introduced, relatively little progress has been achieved via ocean–atmosphere coupled climate models^3,16^.

Among the terrestrial variables, the air temperature is most often considered to be susceptible to solar/geomagnetic activity^6,8,17–20^. However, the responses of other terrestrial variables in addition to temperature to solar/geomagnetic activity have also been considered^4,11,12,21–26^.

The physical mechanisms that govern the behaviour of the terrestrial climate system are not yet sufficiently known. The main causes are the internal instability of the atmosphere and the response of the atmosphere to external factors. The ratio of these two causes remains unknown, despite the increasingly sophisticated modelling in recent studies.

In the present investigation, we use elements of information theory (IT) to test the possible nonlinear influence of external factors, expressed by solar/geomagnetic indices, on terrestrial variables, such as the drought index, discharge in the lower Danube basin, atmospheric circulation indices (Greenland-Balkan Oscillation index (GBOI), North Atlantic Oscillation index (NAOI)), and blocking-type circulation indices over the Atlantic-European region. The influence of the abovementioned terrestrial variables on the discharge in the lower Danube basin was discussed in previous investigations^11,27–29^. Details in the time–frequency domain are obtained by analysing the wavelet transform.

Few works have focused on applying IT to the influence of solar/geomagnetic activity on the earth’s climate. Among the most recent, we mention^30–34^. The IT application in general and especially transfer entropy (TE) is very suitable even in elucidating open problems such as the physical mechanism capable of explaining the connection between the geomagnetic field and climate^30^.

Reviews of wavelet analysis in geoscience and, in particular, for correlations between solar activity and hydrological variables have been published by Labat (2005)^35^ and Fu et al. (2012)^36^. The problem of the signal resolution in the time–frequency domain of the nonstationary processes is assessed by using wavelet analysis^37^.

Details on the wavelet transform method applied to highlight the solar signal quantified by the sunspot number are found in many papers^38–41^.

In several investigations^42–45^ significant solar signals in terrestrial variables have been obtained by applying bandpass filters (BPFs) to isolate certain frequency bands.

The main objective of the present study is to improve our knowledge of the solar/geomagnetic signature in the climate variables that are good predictors of discharge in the lower Danube basin.

The novelty of this study is that we test the influence of two external factors (solar and geomagnetic) by two types of nonlinear methods. First, we consider the influence of the two sources (predictors) on the terrestrial variables (predictands, target) simultaneously and take into account the redundancy due to the connection between the sources by estimating the difference between synergy and redundancy using mutual information. Second, the transfer entropy between each of the predictors and the considered predictands is estimated. The details in the time–frequency domain are explored by using wavelet coherences. We first present the data and methods used and then discuss the results obtained from two simultaneous sources and a target. Because the two sources considered simultaneously produce high redundancy in some cases, we finally separately analyze the signature of each external factor on the terrestrial variables.

## Material and methods

### Datasets

The datasets used in this study consist of variables that characterize terrestrial climate, on one hand, and solar/geomagnetic indices used to describe solar/geomagnetic activity as external factors for the climate system, on the other. The analysis was performed for two time intervals, 1948–2000 (Period I) and 1901–2000 (Period II), taking into account the availability of certain investigated parameters.

#### Terrestrial variables

The influence of solar/geomagnetic activity on climate depends on the spatial scale. We considered the climate variables defined to describe atmospheric circulation from a large scale to a local scale. Thus, for the planetary scale, we use the NAO and GBO indices, and at the Atlantic-European scale, we use blocking indices. For the regional scale, we take into account the drought index for the upper and middle Danube basins; for the local scale, we take into account the Danube discharge at Orsova.

*The planetary scale.* The NAO index, the difference in the normalized sea level pressure (SLP) between Lisbon (Portugal) and Stykkisholmur/Reykjavik (Iceland) was downloaded from http://www.ldeo.columbia.edu/res/pi/NAO/. The GBO index is calculated as the difference in normalized SLP at Nuuk and Novi Sad^46^. The monthly SLP data were obtained from http://rda.ucar.edu/datasets/ds010.1, maintained by the National Center for Atmospheric Research (NCAR). The blocking indices were calculated at the 500 hPa geopotential field (Period I), provided by the *British Atmospheric Data Centre (BADC)* (https://badc.nerc.ac.uk/home/index.html).

Atmospheric blocking circulation is characterized by anticyclonic circulation at high latitudes with cyclonic circulation at low latitudes. Similar to previous investigations^25,29^, the Atlantic-European blocking index (AEBI) is defined on the domain (50°W–40°E; 35°N–65°N). The blocking index for the Atlantic-European region is calculated as the difference between the mean longitudes at 57.5° and 37.5°N. Therefore, a positive value of this index highlights a blocking type circulation and a negative value highlights an atmospheric zonal circulation.

*The regional scale*, Period II. The precipitation and mean temperature in the upper and middle Danube basins from 15 meteorological stations upstream of Orsova were considered. The monthly values of the above variables were downloaded from http://climexp.knmi.nl. The difference between the standardized first principal component of the temperature and precipitation defines a drought index (TPPI).

*The local scale*. The Danube discharge recorded at Orsova station (Q_ORS) is taken as a terrestrial variable. Located between the middle and lower Danube (in Romania), this station represents an integrator of precipitation from the upper and middle basins^11,29^. Data were provided by the National Institute of Hydrology and Water Management, Bucharest, Romania.

#### Solar/geomagnetic data

Solar activity is quantified by solar indices, that represent various solar outputs, such as electromagnetic radiation, solar wind, and interplanetary magnetic fields. Solar indices can be directly related to the sun (direct indices) or related to indirect effects produced by solar activity (indirect indices). Among the direct solar indices, we mention the radio flux at 10.7 cm, F10.7, a physical-based index, and the Wolf sunspot number, a calculated index from the observed sunspot number. The latter is the longest and most commonly used solar activity proxy. Among the effects of solar activity, the geomagnetic activity, which arises from the interaction between the solar wind and the interplanetary magnetic field and the Earth’s magnetosphere, is commonly used. Geomagnetic activity is characterized by geomagnetic indices, which are also considered indirect indices of solar activity.

In the following, external predictors include the Wolf sunspot number (WDC-SILSO, Royal Observatory of Belgium, Brussels, http://www.sidc.be/silso/datafiles) for Period I and the F10.7 index (ftp://ftp.ngdc.noaa.gov/STP/SOLAR_DATA/)) for Period II, together with the *aa* geomagnetic index (http://isgi.unistra.fr/indices_aa.php). Details on the solar radio flux at 10.7 cm are given in Tapping (2013)^47^. The advantages of the F10.7 index over other solar indices are due in part to the fact that it is closely linked to the solar effects on the Earth's atmosphere (Balogh et al. 2014)^48^.

### Methods

#### Information theory elements

Mutual information (MI) is defined as follows:

1 $$MI(X,Y) = H(X) + H(Y) - H(X,Y)$$

where *H(X)* and *H(Y)* represent the information entropies of discrete random variables ***X*** and ***Y***, respectively, and *H(X, Y)* is the joint entropy (Shannon, 1948)^49^.

Synergy and redundancy was considered according to Timme et al. (2014)^50^ as follows:

S-R = Synergy (Y; X_1_,X_2_)−Redundancy (Y; X_1_,X_2_)

2 $$S - R = MI\,\;(X_{1} ,X_{2} ;Y) - MI\,\;(X_{1} ,Y) - MI\,\;(X_{2} ,Y)$$

The contribution of predictors (*X*_*1*_*, X*_*2*_) to predictand (Y), which includes a reduction in redundancy, is obtained by simultaneous analysis of synergy and redundancy, given by Eq. (2). A negative value implies that the redundant contribution is greater in magnitude than the synergetic contribution.

The total correlation (TC) in the mutual information terms for three variables (Timme et al., 2014)^50^ is as follows:

3 $$TC = MI(X_{1} ;X_{2} ) + MI(X_{1} ,X_{2} ;Y)$$

where TC is a measure of the total information between all variables.

As shown in Bennett et al. (2019)^51^, a method for quantifying the transfer of information from one variable to another was developed by Schreiber (2000)^52^ and has been applied in many investigations^53–55^. According to Timme and Lapish (2018)^56^, the transfer entropy (TE) using conditional mutual information is given as follows^52^:

4 $$TE(X \to Y) = MI(Y_{future} ;X_{past} /Y_{past} )$$

Useful examples and discussions on both the theoretical and practical applications of IT can be found in^56^. For simplicity, if we note *Y*=*Y*_*future*_, *X*_*1*_= *X*_*past*_ and *X*_*2*_= *Y*_past_, Eq. (4) is written in terms of entropy according to Kay et al. (2017)^57^ as:

5 $$MI(Y;X_{1} /X_{2} ) = H(Y,X_{2} ) + H(X_{1} ,X_{2} ) - H(X_{2} ) - H(Y,X_{1} ,X_{2} )$$

### Wavelet coherence

In the present study, we applied wavelet analysis to highlight the repartition in the time–frequency domain of the coherence between two or more variables. Wavelet coherence (WTC) for two variables is a measure of the intensity of the covariance of the two series in time–frequency space. *Coherence* is defined as the square of the cross-spectrum normalized by the individual power spectra, which produces a quantity between 0 and 1 and measures the cross-correlation between two time series as a function of frequency. Details are found in papers by Jevrejeva et al. (2003)^58^ and Torrence and Webster (1999)^59^.

In the case of two variables, one of them is either the geomagnetic activity index or the solar flux/Wolf sunspot number. For the coherence of several variables, we consider the two predictors, solar and geomagnetic indices, simultaneously along with one of the terrestrial variables.

The wavelet analysis in this study assumes that the red noise characteristics are modelled as a first-order autoregressive process AR(1)^36,60,61^. The statistical significance level of the wavelet coherence in comparison with red AR(1) noise is estimated using Monte Carlo methods^58,61^. All parameters for WTC were calculated using the Matlab procedure (http://www.glaciology.net/wavelet-coherence) provided by Grinsted et al. (2004)^61^. Multiple wavelet coherence was performed based on the Hu and Si (2016)^62^ algorithm.

## Results and discussion

### One target and two simultaneous sources

Here, the target is one of the terrestrial variables and the sources include the solar/geomagnetic indices. We assess the difference between synergy and redundancy (S-R) and the total correlation (TC) for the two time intervals. The terrestrial variables were the TPPI, Q_ORS, GBOI and AEBI for Period I and the TPPI, Q_ORS, GBOI and NAOI for Period II. We performed separate analyses on each season to highlight the simultaneous influence of the two sources on each of the predictands. A detailed interpretation of the relation between the synergy and redundancy for a system with 3 variables, i.e., one target and two predictor variables, is given by Ince (2017)^63^. A higher TC and S-R (positive) indicate that the information provided by the two simultaneous predictors has a greater ability to reduce the uncertainty of the predictand.

### Period I

**Figure 1:**
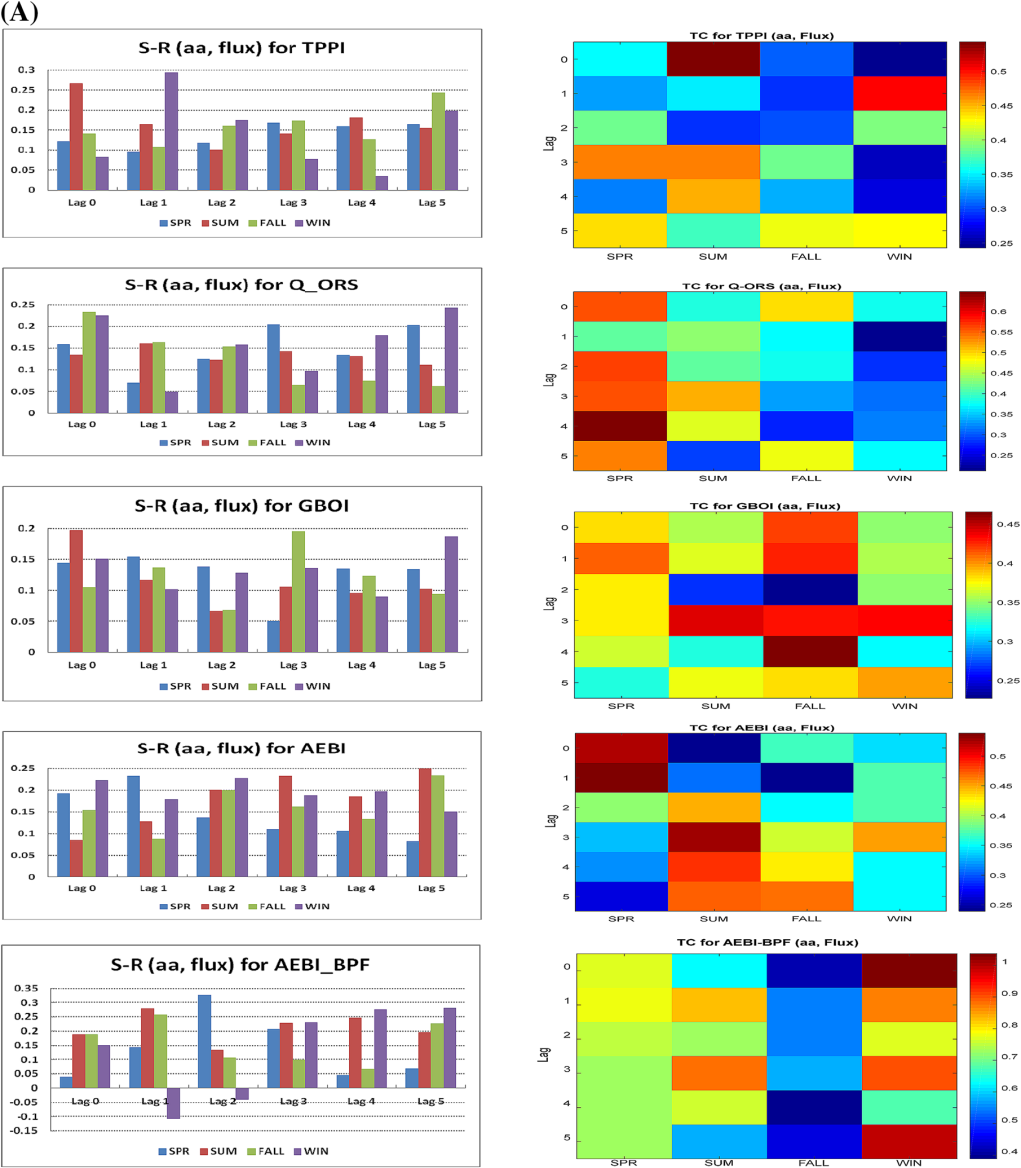

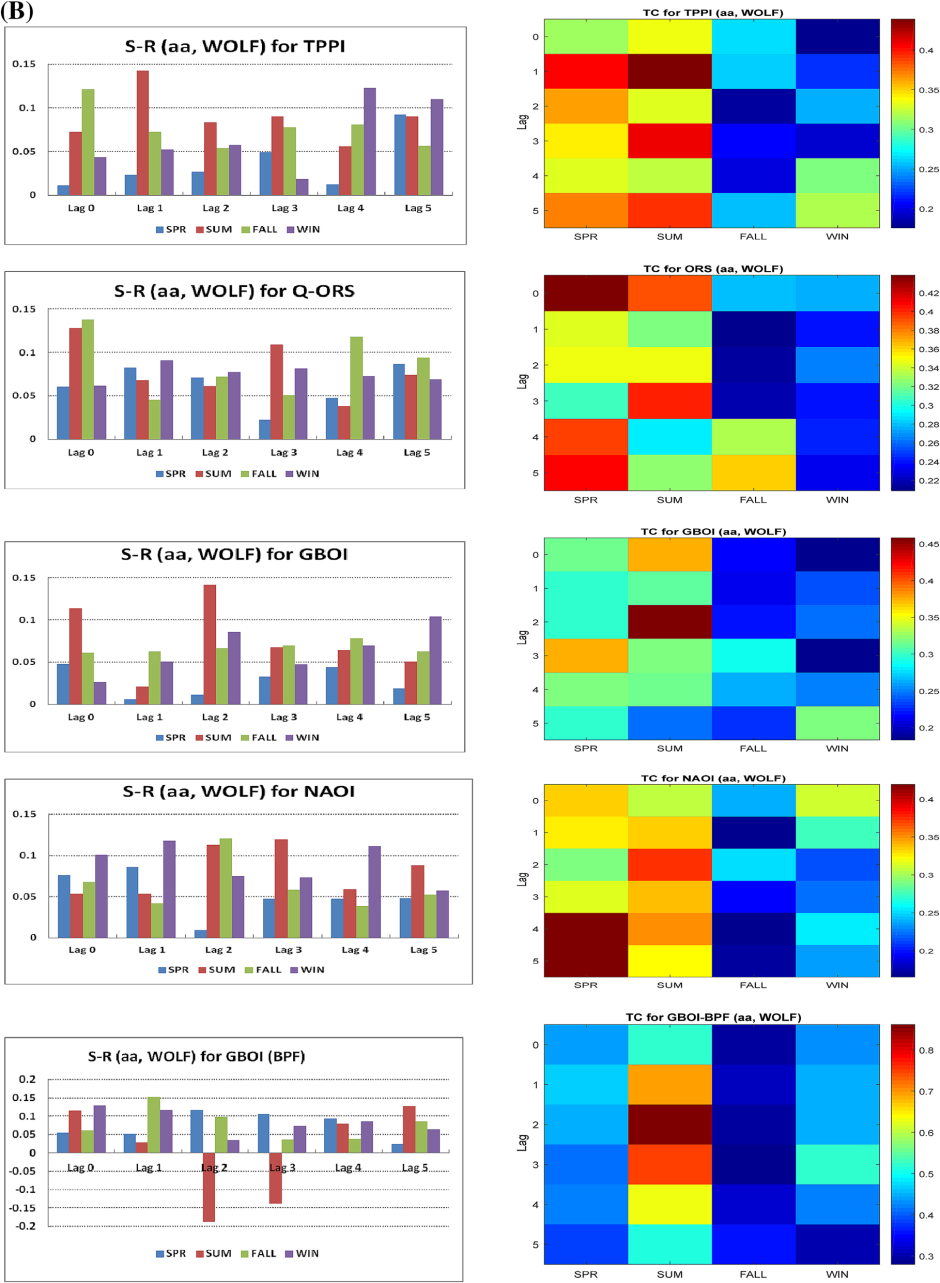
(**A**) The difference between synergy and redundancy (S-R) (left side) and the corresponding total correlations (TC) (right side), for Period I, in case of two predictors taken simultaneously (*aa* and solar flux) and climate variables. From top to bottom, TPPI, Q_ORS, GBOI and AEBI. The bottom panels represent (S-R) and TC for BPF data in case of two predictors and AEBI. (**B**) The difference between synergy and redundancy (S-R) (left side) and the corresponding total correlations (TC) (right side), for Period II, in case of two predictors taken simultaneously (*aa* and Wolf number) and climate variables. From top to bottom, TPPI, Q_ORS, GBOI and NAOI. The bottom panels represent (S-R) and TC for BPF data in case of two predictors and GBOI.

For the time interval 1948–2000, Fig. 1A shows the S-R values for the analysed predictand variables on the left side and the corresponding TC values on the right side for each season. The influence of the two sources (*aa* and F10.7) on terrestrial variables was calculated for lags from 0 (simultaneously) to 5 (years). In all these cases, the data are unfiltered. Bandpass filtering (BPF) in the frequency band of 9–15 years (details in Mares et al. (2016)^25^) for AEBI is also shown in the bottom panels of Fig. 1A. Although the TC is relatively high for the winter season, at lags 1 and 2, the difference between synergy and redundancy is negative, thereby negating the concomitant use of the two predictors.

**Figure 2:**
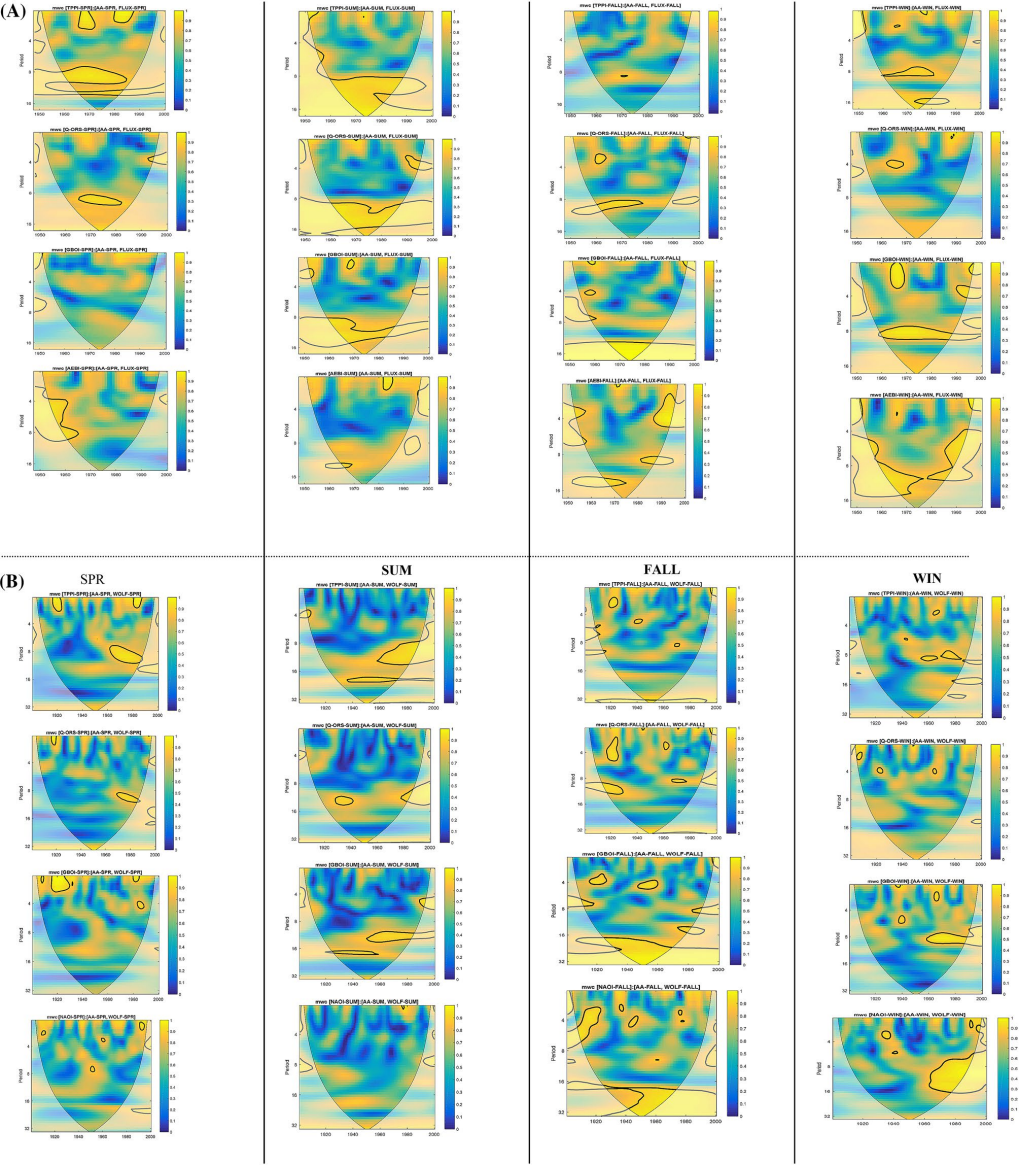
(**A**) Multiple wavelet coherence (MWC) between *aa* and solar flux for Period I. (**B**) Multiple wavelet coherence (MWC) between *aa* and Wolf number for Period II

Figure 2A represents the simultaneous multiple wavelet coherence (MWC) obtained by considering the two predictors together with one of the terrestrial variables, which corresponds to lag 0 (simultaneous connections), for the unfiltered data shown in the first 4 panels of Fig. 1A.

The analysis of the results from the two figures for lag 0 indicates that the relatively high positive values of S–R are associated with a significant MWC for multiple wavelengths in summer in the case of the TPPI, in summer and winter for GBOI, and in winter for AEBI. For lags from 1 to 5 (Fig. 1A), taking into account S-R and then TC, there is an influence of the two sources in the case of the TPPI in winter at lag 1, for the discharge at Orsova in winter at lag 5 and for GBOI in autumn at lag 3. In the case of AEBI, the action of the two predictors is noticeable in spring at lag 1 and in summer at lag 3.

### Period II

The S-R values and corresponding TC at lags from 0 to 5 years between the two predictors and a target from 1901 to 2000 are presented in Fig. 1B. The results differ depending on the season and the target variable. Taking into account both S-R and TC, for the TPPI drought index, the most significant contribution of the two sources is during summer at lag 1. However, for the Danube discharge at Orsova, the TC has the highest value at lag 0 during spring, and due to the higher S-R values during summer and autumn, we consider that the best contribution of the two predictors is in summer based on the TC. Zanchettin et al. (2019)^64^ analysed discharge time series of major European rivers, including the Danube (at Bratislava), and found significant fluctuations for the Po and Danube rivers during summer and winter. As shown in Zanchettin et al. (2008)^65^, summer fluctuations for some states of the hydrological regime in Europe can be attributed to solar activity. Regarding the NAOI, the analysis of both S-R and TC values indicates that the two sources have an effect during summer at lags 2 and 3.

In the case of GBOI, for this time interval, we use both unfiltered and filtered data by BPF (9–15). A comparison of the results shows that filtering greatly changes the information provided by the two predictors. Thus, for the case of the unfiltered data, the highest values of S-R accompanied by high TC are during summer at lag 2, whereas for the filtered data, the situation is exactly the opposite regarding S-R. In this case, the S-R value is negative, even if the TC value is the highest. Therefore, the filtered data introduce spurious total correlations, such as in the case of linear correlations, which in turn lead to high redundancy, meaning that the considered predictors cannot be used together to help improve the estimation of the predictand. In this regard, we note that the study of Mares et al. (2016)^25^ focused on the linear correlations between filtered and unfiltered data.

The analysis of Fig. 2B, the multiple wavelet coherence between *aa*, Wolf number and terrestrial variables at lag 0, does not clearly show coherence for several wavelengths. It can be supposed that for GBOI during summer, significant coherence is observed in the band corresponding to periods 9–15, which can be associated with a positive S-R value of 0.10 in the case of filtered data. Additionally, for the winter season, significant coherence is observed among the *aa*, Wolf number and NAOI after the 1970s, for which the estimated S-R has a positive value of 0.10. These results are consistent with those obtained by Bochnícek et al. (2012)^22^, who considered the concomitant action of solar and geomagnetic forcings on winter atmospheric circulation using a nonlinear method, namely, that of *composite maps*.

Significant correlations between solar/geomagnetic activity and NAOI after the 1970s were also found by Thejll et al. (2003)^27^.

### One source and one target

As a complementary investigation to the one in the previous paragraph, when we considered two simultaneous sources that could influence a target, we separately considered either a geomagnetic index or solar index as a source for Period I and Wolf number as a source for Period II, which could produce a signal in one of the terrestrial variables. The testing of the connection between terrestrial variables and external factors was performed using the TE estimated according to Eq. (4) and detailed in Eq. (5). The results obtained in this way can improve situations in which the two predictors simultaneously produce a high redundancy. For example, Fig. S1 in the *Supplementary Information* shows that for certain months and certain lags, the TE between *aa* and solar flux or between *aa* and Wolf number have high values, especially for the filtered data. In these cases, high connectivity is observed between these variables, which explains low or even negative values for the difference between synergy and redundancy when they are considered together as predictors.

### Period I

**Figure 3:**
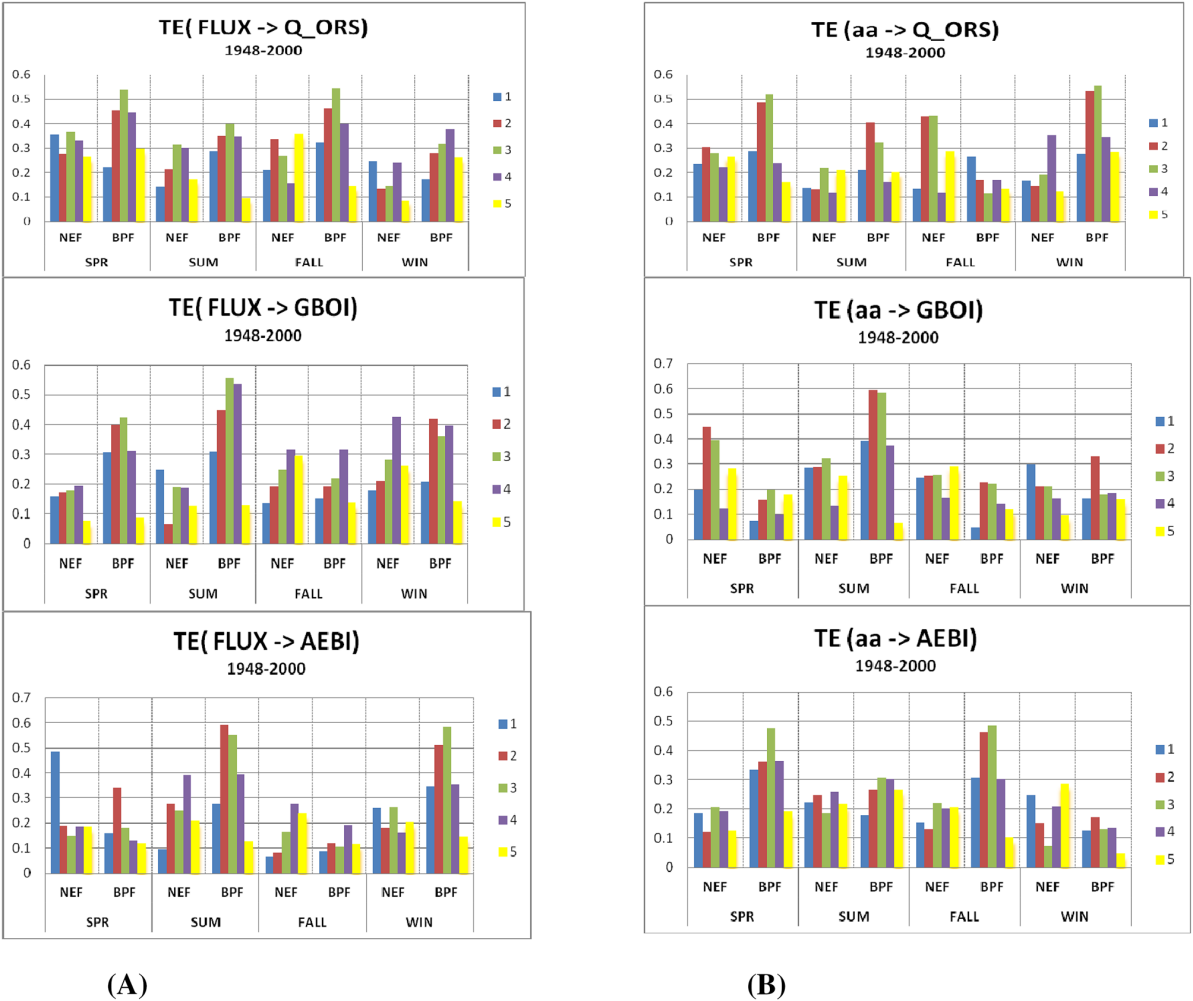
(**A**) The transfer entropy (TE) from solar flux to Q_ORS, GBOI and AEBI, with lags from 1 to 5 years, in case of unfiltered (NEF) and filtered (BPF) time series, for each season in the Period I. (**B**) The TE from *aa* geomagnetic index to Q_ORS, GBOI and AEBI, with lags from 1 to 5 years, in case of NEF and BPF time series, for each season in the Period I.

Figure 3A,B display the TE values between the solar flux and Q_ORS, GBOI and AEBI and between the geomagnetic index *aa* and the terrestrial variables, respectively. The highest values of TE are found for the filtered data (BPF) with delays of 2 or 3 years compared to the solar flux, which is more obvious in the summer and winter seasons.

In general, the TE values that represent the transfer of information from *aa* to the terrestrial variables are lower than those corresponding to the transfer from the solar flux to terrestrial variables. The exception is winter when the TE from *aa* =  > Q_ORS for the filtered data from lags 2 and 3 is higher than that from the flux to Q_ORS. The same situation is observed for filtered data in the case of TE from *aa* to AEBI at lags 1–3 during spring and fall.

Considering the importance of the Danube discharge at Orsova, during the spring, it can be observed (Fig. 3A) that for both unfiltered and filtered data, the TE values from the flux to discharge are relatively high, especially for the third lag.

In Fig. S2 (*Supplementary Information*), the WTC between unfiltered solar flux and discharge for spring (1948–2000) is shown. This figure shows that the two time series are coherent in the period of 10–12 years (1965–1975), with a 95% confidence level. Because the arrows are not horizontal, there is a lag between the two time series. Taking into account that the two series have a negative correlation, the arrows in Fig. S2 indicate that the solar flux leads to ¼ period discharge, namely, approximately 3 years. From the above results, we can expect that at approximately 3 years after a maximum (minimum) solar activity, the spring discharge will be lower (higher).

Peaks of some river discharge power spectra associated with solar variability were found in the investigations^65–68^. Although our results are obtained from relatively short time series, they are consistent with the results found by Peña et al. (2015)^69^. These authors investigated summer floods in Switzerland for more than 300 years and concluded that a high frequency of flooding is related to the solar activity minimum and that a summer flood damage index shows a significant component with a frequency corresponding to 10–12 years.

**Figure 4:**
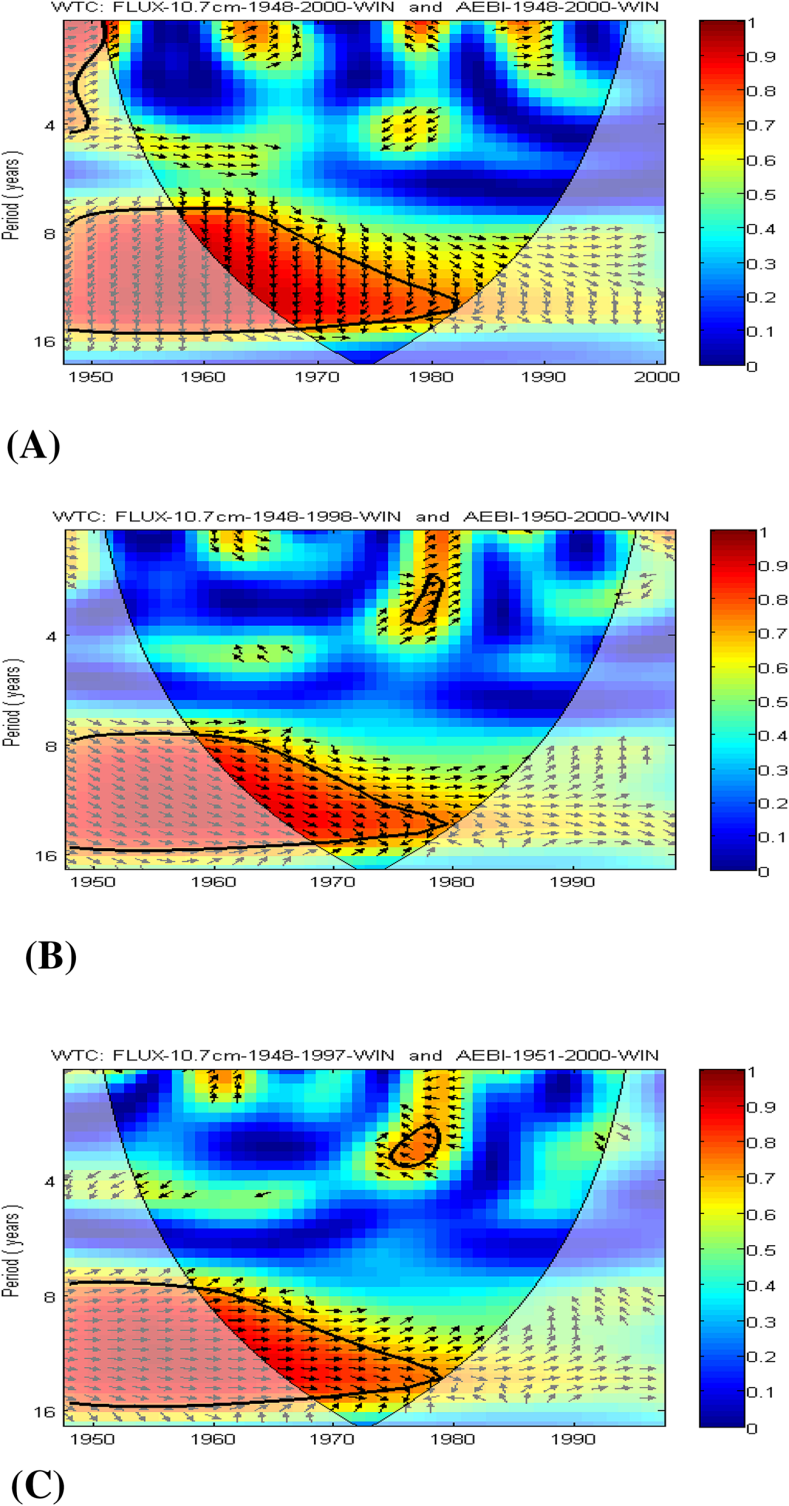
Wavelet coherence (WTC) between solar flux and AEBI during winter, for Period I: (**A**) unfiltered time series; (**B**) 2 years lag; (**C**) 3 years lag.

Regarding the entropy transfer from solar flux to AEBI, the high values of TE in winter for the BPF data (Fig. 3A) can be explained by the results of the coherence obtained by applying the wavelet transform (Fig. 4). There is good coherence between the solar flux and AEBI in the band corresponding to periods of 9–15 years, and at lag 3, the two variables are also in phase. From Fig. 4, we find from the WTC representation that this link between the solar activity quantified by the solar flux and the atmospheric circulation of blocking type over the Atlantic European region in winter is not stationary over time. Thus, in Fig. 4A, where the WTC between the two time series (unfiltered) and without any lag is represented, the two time series have a significant coherence for the 8–15 year period band between 1948 and 1983. However, taking into account that the area located outside the cone of influence must be regarded with caution, this coherence is significant between 1960 and 1983. The arrows in Fig. 4A are not horizontal; therefore, they indicate that there is a lag between the two series. Because the two initial series have a positive correlation, the arrows indicate that the first series, which corresponds to the solar flux, drives the second AEBI series, with a quarter of a period meaning approximately 3 years. Figure 4B,C show WTCs for 2- and 3-year lags, respectively, between the solar flux and the blocking indices. These last figures show that the areas with significant coherence are similar, with a slight intensity of coherence if the lag between the solar flux and AEBI is 3 years.

**Figure 5:**
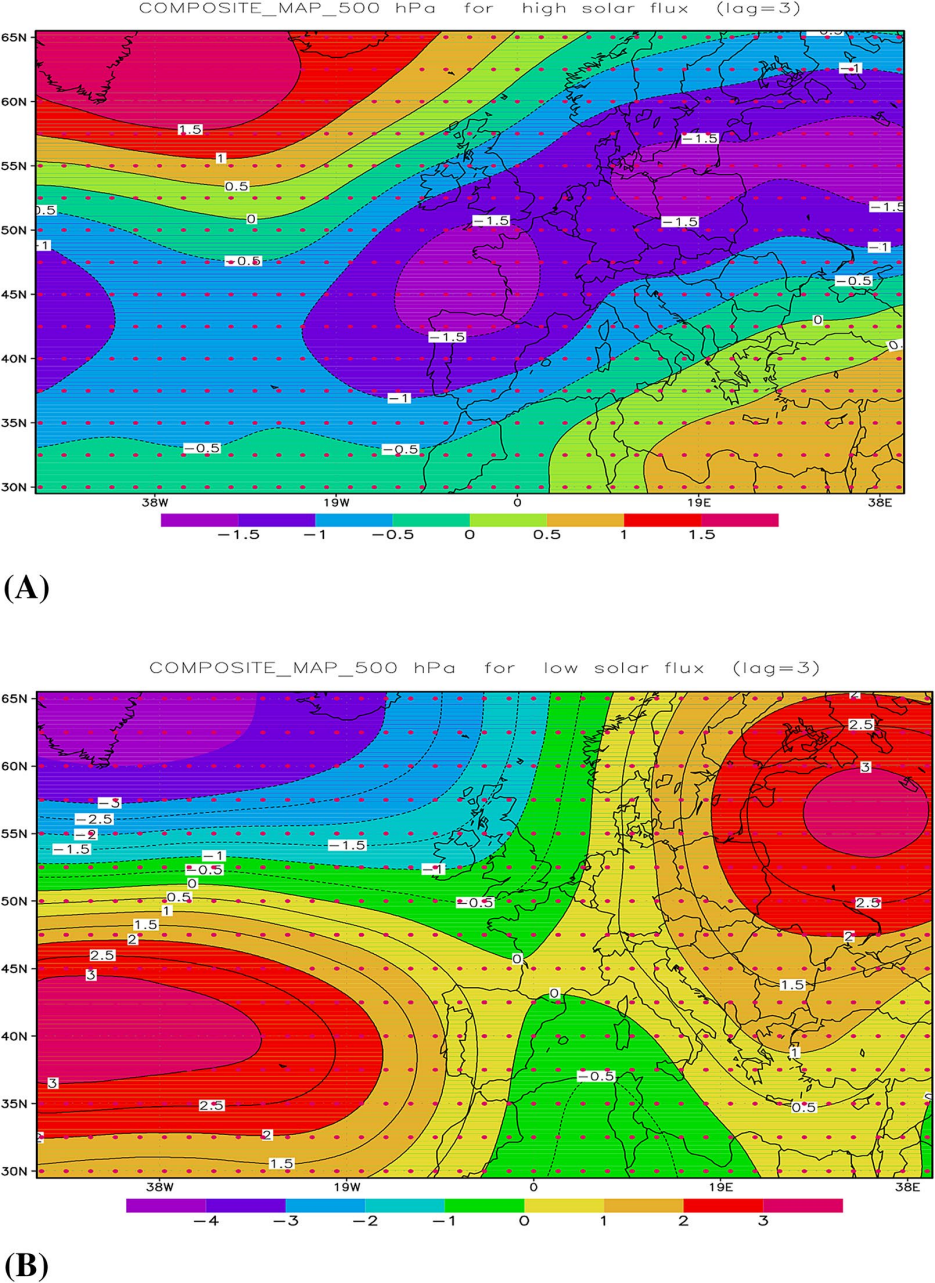
Composite maps for winter anomalies at 500 hPa, corresponding to the solar flux leading with 3 years the geopotential for two cases: (**A**) high flux; (**B**) low flux.

Considering the results obtained by Mares et al. (2016)^25^, approximately 2–3 years after the maximum (minimum) solar activity, the atmospheric circulation of the blocking type is enhanced (weakened) during winter over the Atlantic-European region. This result is also confirmed by *composite maps* at 500 hPa for the winter season, and when high solar flux (Fig. 5A) and low solar flux (Fig. 5A) are observed, the geopotential field that lags the solar flux by 3 years is considered. Here, we define high-flux cases as years in which the standardized values of the solar flux are greater than 1 and low-flux cases as years in which the standardized flux values were less than − 1. In the first case (Fig. 5A), the *composite map* defines a positive blocking index, and in the second case (Fig. 5B), the blocking index is negative.

The advantages of applying nonlinear and nonstationary techniques are also highlighted by comparing the results obtained here with those obtained by applying the analysis of linear correlations. For example, Fig. S3a in the *Supplementary Information* shows that for the unfiltered and filtered data, the Pearson correlation coefficient between the solar flux and the AEBI does not show results with different statistical significance because the connection between the two variables is nonstationary and nonlinear. The graph in Fig. S3b indicates good coherence between the solar flux and AEBI for the period 1965–1985, which is consistent with the WTC in Fig. 4B.

### Period II

**Figure 6:**
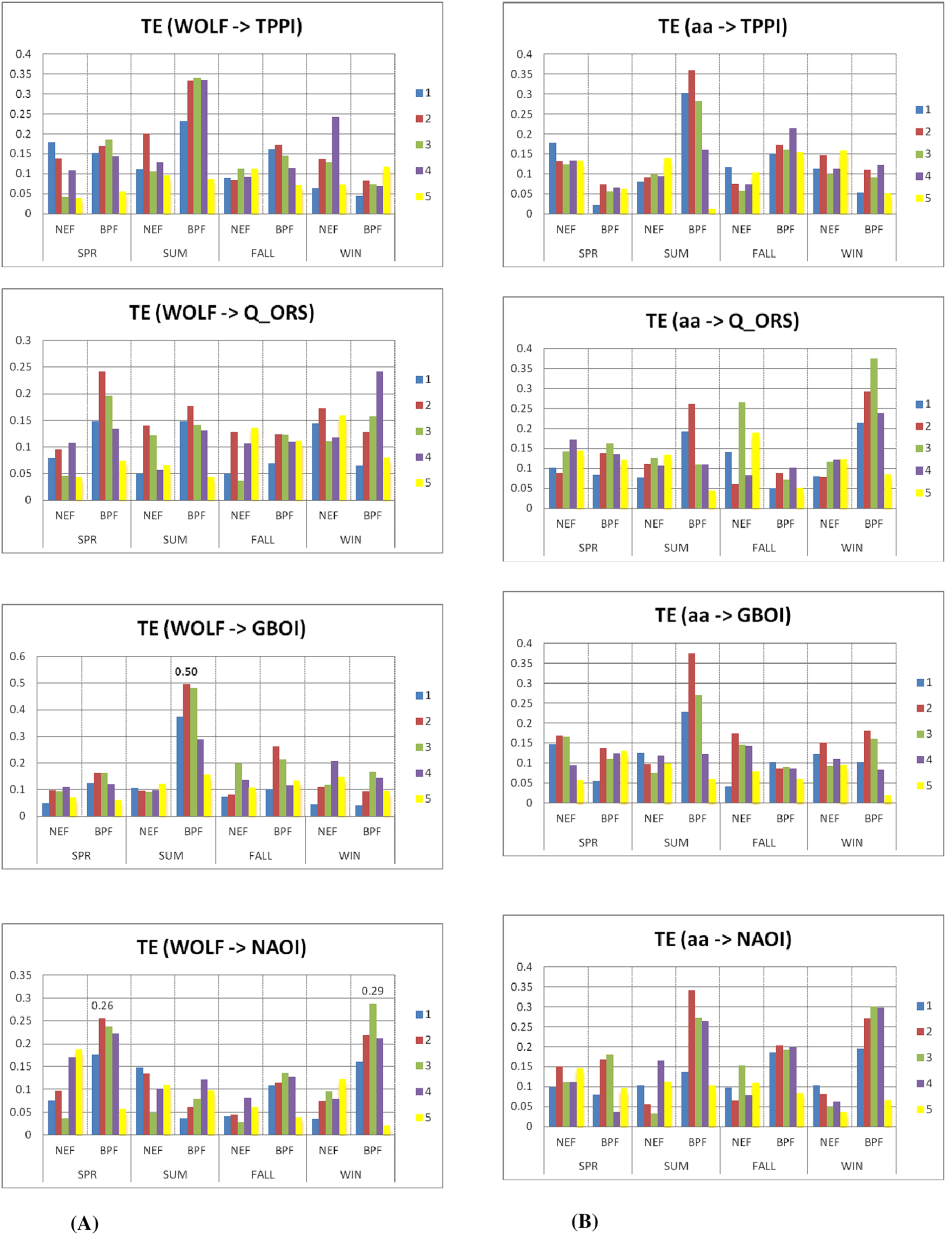
(**A**). The transfer entropy (TE) from Wolf number to TPPI, Q_ORS, GBOI and NAOI, with lags from 1 to 5 years, in case of unfiltered (NEF) and filtered (BPF) time series, for each season in the Period II. (**B**) The transfer entropy (TE) from *aa* geomagnetic index to TPPI, Q_ORS, GBOI and NAOI, with lags from 1 to 5 years, in case of unfiltered (NEF) and filtered (BPF) time series, for each season in the Period II.

Figure 6A shows the TE values corresponding to the transfer entropy from solar activity, which is represented by the Wolf number, and four terrestrial variables, the TPPI, Q_ORS, GBOI and NAOI, for each season, with lags from 1 to 5, for both unfiltered and filtered data. Figure 6B shows the TE values from geomagnetic index *aa* using the same terrestrial variables. The TE values are dependent on the season, the target variable and the lags. The highest TE value of 0.50 was found for the transfer entropy from the Wolf number to GBOI during summer at lag 2 using the filtered data. Additionally, the transfer from *aa* to the corresponding GBOI is relatively high, with a TE of 0.37; however, because the S-R value of the two sources is negative (Fig. 6B), we cannot consider the simultaneous influence. Therefore, we will focus on the influence of the solar signal on the GBOI during summer.

An explanation of the physical mechanism of correlations with certain lags between solar activity and climate variables can be found in previous studies^70–72^. A possible response of the atmospheric circulation GBO index to solar variability with a delay of 2–3 years is due to ocean–atmosphere interactions, as described by Thiéblemont et al. (2015)^72^, who analysed the solar signal in NAOI. The authors proposed a new synchronization mechanism that combines air–sea interaction processes and solar-induced stratospheric dynamics modulation to simulate the observed solar influences on the North Atlantic climate using a coupled ocean–atmosphere model under two versions. As shown in Chen et al. (2015)^14^, possible mechanisms have been proposed for this lagged response, including a delayed response based on the extended memory of ocean heat content^71,73^.

In the present investigation, we did not analyse the relationship between solar flux and the NAOI. According to the transfer entropy from solar activity, which is expressed as the Wolf number, to the NAOI, the TE has the highest values (winter) for filtered data (9–15) for delays from 2–3 years (Fig. 6A), which is consistent with the results obtained by Thiéblemont et al. (2015)^72^.

Bochníček and Hejda (2005)^22^ found that during winter, geomagnetic activity is more closely associated with the NAOI than solar activity, and they provided a possible physical mechanism for this result. The analysis of TE values in Fig. 6A,B shows that in winter with lags from 1 to 5 years, the values of the transfer entropy from the geomagnetic index *aa* to the NAOI are slightly higher than the TE from the Wolf number to the NAOI in the case of filtered data.

Additionally, the correlation analyses by linear correlation coefficients shown in Table S1 in the *Supplementary Information* indicate that for the NAOI, the most significant values of the link between the Wolf number and NAOI were obtained for delays of 2 and 3 years for winter.

In addition, for the other variables analysed in relation to solar or geomagnetic activity, the values of the Pearson or Kendall correlation coefficients can be compared with the TE values from Fig. 6. Thus, in the case of the drought index used in this study, good concordance exists between the results obtained by linear methods, i.e., the correlation coefficients between the TPPI and solar/geomagnetic activities (Table S1), and those obtained by the nonlinear TE method are represented in the graphs in Fig. 6A,B. Both the correlation coefficients and TE indicate a significant link during summer between the TPPI and Wolf number and between the TPPI and the geomagnetic index (for filtered data). The latter connection is slightly higher than the connection with the Wolf number at lag 2, which is highlighted by both methods and represents is an important result because European countries located in the Carpathian region are affected by drought episodes and present greater vulnerability to climate change^74^.

Therefore, the investigation of both internal (atmospheric circulation quantified by large-scale atmospheric indices) and external forcings on the earth’s climate system tested in this study led to an improvement in the estimation of drought/wet episodes in the Danube basin and the adjacent area.

Details on the methods for testing the nonlinear link between solar/geomagnetic indices and some terrestrial variables are given in the *Supplementary Information (Section II)*.

Related to external forcing, the solar signal acts both directly on hydroclimatic variables and through modulators, such as the quasi-biennial oscillation (QBO). Therefore, nonlinearities alone can produce noticeable climate extremes consistent with observations. However, even for quite strong solar modulation of the model climate, nonlinearities are capable of intermittently disrupting this modulation^75^. More recent studies^76^ lead us to postulate that the modulator of the solar signal for hydroclimatic phenomena is the anomalous QBO that amplifies the solar signal in the initial moments and days of phase changes from west to east. This phenomenon might be caused by the angular momentum related to the QBO, which has maximum action at long distances (for the lower troposphere) at the time of transition from one phase to another.

Details of the authors^25^ previous results on the role of QBO are given in Supplementary Information (Section III-A).

Interesting results are also obtained by linear methods on certain dominant factors, such as NAO, which determine the large-scale atmospheric variability in Europe^77^. But this happens when there are no large deviations from linearity in the connection of phenomena.

## Conclusions

We have shown that the impact of solar/geomagnetic activity on the hydroclimate is significant and can be discriminated from cause to effect. The chosen method is a nonlinear method that quantifies the synergistic cumulative impact of several factors by removing possible redundancies, thus allowing for the discrimination at multiple spatiotemporal scales.

The impact of solar/geomagnetic activity on climate variables in the Danube basin was first tested using elements of information theory. Based on the difference between synergy and redundancy (S-R) calculated both simultaneously and with delays from 1 to 5 years in the terrestrial variables, the possibility of using both solar and geomagnetic indices as sources for reducing the uncertainty of one of the terrestrial variables has been highlighted. We also calculated the total correlation (TC) based on mutual information of three variables (two sources and one target) associated with each case for both unfiltered and filtered data in the band corresponding to the periods of 9–15 years, which separate analyses for each season. Although the highest values of TC were generally obtained for the filtered data, because solar and geomagnetic indices are closely correlated and highly redundant (negative S-R), we analysed the case with one source and one target.

The nonlinear analysis of one source and one target was performed using the transfer entropy (TE). The obtained results differ depending on the time of year and the analysed variables. Mainly, the TE values from the *aa* to the terrestrial variables are lower than the TE values from solar indices. Moreover, we found cases in which the TE from the *aa* to TPPI, Q_ORS and NAOI is slightly higher than the TE from the Wolf number to the corresponding variables during the summer and winter seasons for Period II, 1901–2000.

For Period I, 1948–2000, where the solar activity was quantified by the solar radio flux, the most significant results are for the lower Danube discharge and the atmospheric circulation over the Atlantic-European region. In the first case, the signal of the solar flux in the Danube discharge is significant during the spring season with a delay of 3 years. For the AEBI, after 2–3 years, the wavelet coherence during the winter season is considered inconclusive after the 1980s. The impact of solar activity on the analysed climate variables is not readily apparent in recent decades, and the climate variability might be explained by the increase in the effect of greenhouse gas, which has been shown by many authors.

Our results suggest that significant solar signals occurred in the time intervals when the Wolf number was higher in solar cycles 18–22. However, the mechanisms by which these geomagnetic/solar signals influence terrestrial variables are still unclear.

The approach that connects solar activity and different hydroclimatic variables by nonlinear methods based on informational entropy highlights not only the connections from cause to effect but also the relevant mechanisms in the solar signal circuit to the Earth's surface.

The present study has provided additional information on the signature of solar/geomagnetic variability in terrestrial variables in the Danube basin and in climate indices over the Atlantic-European region. Although some of the findings are not conclusive, the significant findings can be used for indicator purposes. Together with the climate predictors found in our previous papers^25,27–29^, these findings contribute to improving estimates of the effect of climate variability on lower Danube discharge.

## Supplementary Information

Supplementary Information.
