# Supplementary material for: Assessing the solar variability signature in climate variables by information theory and wavelet coherence
Source: Sci Rep. 2021 May 31;11:11337. doi: 10.1038/s41598-021-90044-6 (PMC8167174; doi:10.1038/s41598-021-90044-6)
Supplement: Supplementary file 1 — Supplementary Information. [file 41598_2021_90044_MOESM1_ESM.doc]

***Supplementary Information***

**Assessing the solar variability signature in climate variables by information theory and wavelet coherence**

***Ileana Mares^1*^, Venera Dobrica^1^, Constantin Mares^1^ & Crisan Demetrescu^1^***

^1^ Institute of Geodynamics, Romanian Academy, Bucharest, Romania. ^*^ Correspondence to I.M. (email: [ileana_mares@geodin.ro](mailto:ileana_mares@geodin.ro)

Index

1. **LINEAR CORRELATIONS AND WAVELET ANALYSIS............................1-3**
2. **ON THE TIME SERIES NONLINEARITY................................................... 4-8**
3. **POSSIBLE MECHANISMS OF THE SOLAR/ GEOMAGNATIC**

**SIGNATURE ON THE TERRESTRIAL VARIABLES ............................... 8-12**

1. **LINEAR CORRELATIONS AND WAVELET ANALYSIS**

| *Corelatiions with WOLF number* | | | | | | | | |
| --- | --- | --- | --- | --- | --- | --- | --- | --- |
| *Variable* | *lag* | *Season* | *r* | *t* | $\tau$ | *p* | *n_e_* | *CL* |
| TPPI (9-15) | **1** | **SPR** | **0.525** | **2.288** | **0.375** | **0.000** | **16** | **95%** |
|  | **1** | **SUM** | **0.489** | **2.137** | **0.328** | **0.000** | **17** | **95%** |
|  | **1** | **FALL** | **0.597** | **3.023** | **0.444** | **0.000** | **19** | **99.5%** |
|  | **2** | **SUM** | **0.645** | **3.204** | **0.445** | **0.000** | **16** | **99.5%** |
|  | **3** | **SUM** | **0.611** | **2.929** | **0.425** | **0.000** | **16** | **99%** |
| GBOI(9-15) | 1 | SPR | 0.391 | 1.661 | 0.276 | 0.000 | 17 | ~90% |
|  | 2 | SPR | 0.401 | 1.712 | 0.278 | 0.000 | 17 | ~90% |
|  | **2** | **SUM** | **-0.544** | **2.440** | **-0.407** | **0.000** | **16** | **95-98%** |
|  | **3** | **SUM** | **-0.690** | **3.657** | **-0.539** | **0.000** | **17** | **99.5%** |
|  | 3 | FALL | 0.418 | 1.759 | 0.280 | 0.000 | 17 | 90% |
| NAO(9-15) | **2** | **WIN** | **0.515** | **2.403** | **0.336** | **0.000** | **18** | **~98%** |
|  | **3** | **FALL** | **0.519** | **2.438** | **0.355** | **0.000** | **18** | **~98%** |
|  | **3** | **WIN** | **0.552** | **2.654** | **0.408** | **0.000** | **18** | **98%** |
| Q_ORS(9-15) | 1 | SPR | -0.438 | 1.806 | -0.305 | 0.000 | 16 | 90% |
|  | **1** | **SUM** | **-0.495** | **2.108** | **-0.333** | **0.000** | **16** | **95%** |
|  | 1 | FALL | -0.431 | 1.749 | -0.293 | 0.000 | 15 | 90% |
|  | **2** | **SPR** | **-0.504** | **2.156** | **-0.347** | **0.000** | **16** | **95%** |
|  | **2** | **SUM** | **-0.589** | **2.682** | **-0.405** | **0.000** | **16** | **98%** |
|  | **3** | **SUM** | **-0.507** | **2.167** | **-0.343** | **0.000** | **16** | **95%** |
| ***Correlations with aa*** | | | | | | | | |
| **TPPI(9-15)** | **1** | **SUM** | **0.885** | **7.085** | **0.699** | **0.000** | **16** | **99.9%** |
| **TPPI(9-15)** | **2** | **SUM** | **0.730** | **3.962** | **0.545** | **0.000** | **16** | **99.8%** |
| **TPPI(9-15)** | **3** | **SUM** | **0.375** | **1.519** | **0.269** | **0.000** | **16** | **95%** |
| Q_ORS(9-15) | **1** | **FALL** | **-0.589** | **2.585** | **-0.412** | **0.000** | **15** | **95-98%** |
|  | **2** | **SUM** | **-0.644** | **3.036** | **-0.461** | **0.000** | **15** | **99%** |
|  | 2 | FALL | -0.470 | 1.883 | -0.310 | 0.000 | 14 | ~90% |
|  | **2** | **WIN** | **-0.571** | **2.550** | **-0.405** | **0.000** | **15** | **95-98%** |
|  | 3 | SUM | -0.367 | 1.437 | -0.248 | 0.000 | 15 | 80-85% |
|  | **3** | **WIN** | **-0.573** | **2.603** | **-0.401** | **0.000** | **16** | **98%** |

Table S1. The correlations with the lags from 1 to 3 between solar/geomagnetic indices (1901-2000) and terrestrial variables. Confidence level (CL), variables filtered by band pass filtered (BPF). *r*- Pearson correlation coefficient, *t*- the values of test *t*, $\tau$**- Kendall correlation coefficient, p -** significance p-level, *n_e_* is the effective number*.*

*The values corresponding to the high confidence level are marked in red.*


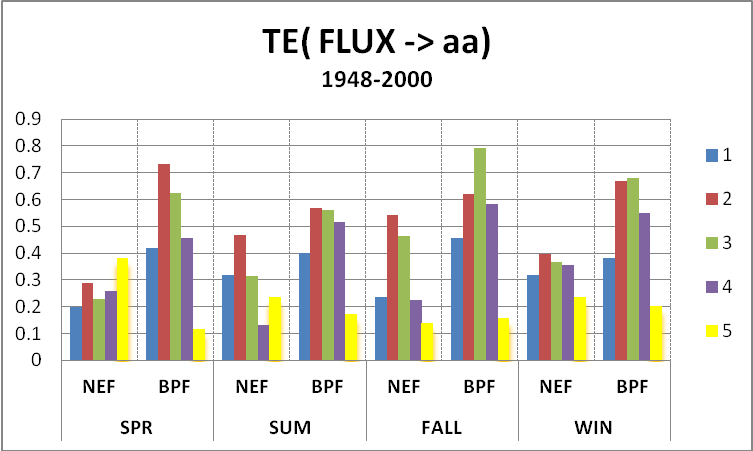


a)


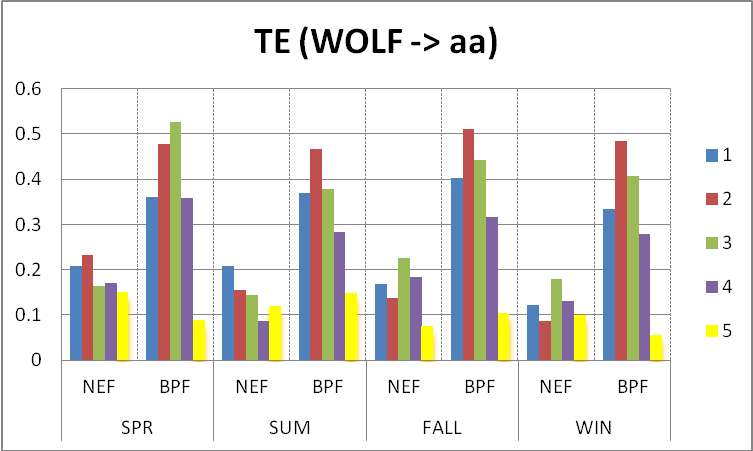


b)

*Figure S1*. Transfer entropy (TE) from

1. solar flux to *aa* (1948-2000) and
2. Wolf number to *aa* (1901-2000).

NEF – unfiltered data; BPF- band pass filter (9-15)

*Figure S2.* Wavelet coherence (WTC) between unfiltered solar flux and the discharge time series during spring (1948-2000).


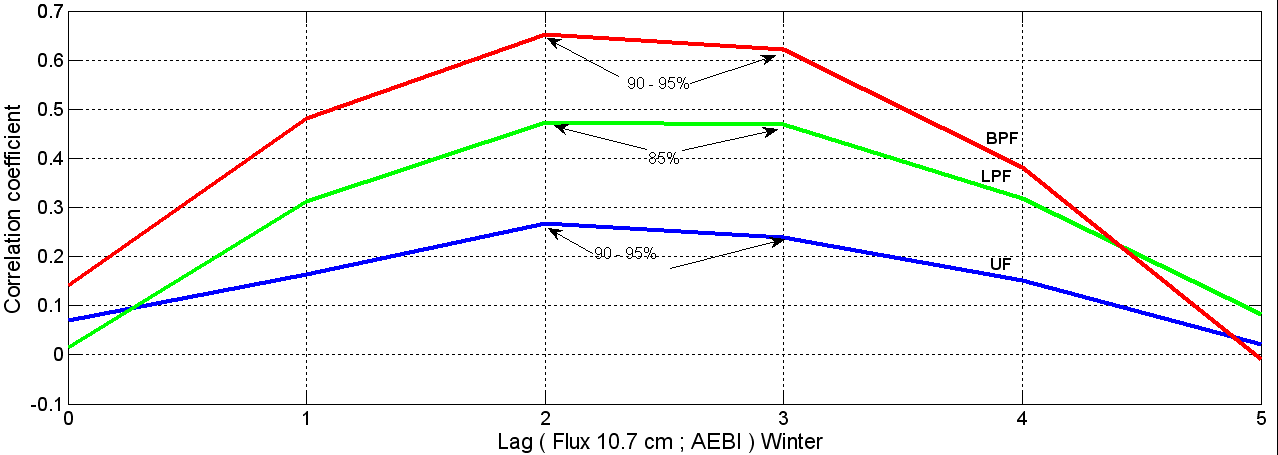


a)

b)

*Figure S3*. Solar signal in the blocking index over Atlantic-European (AEBI), during winter (1948-2000).

1. Correlation coefficients, between solar flux **F10.7** and AEBI with the lags 0-5, in case of : unfiltered (UF), smoothing by low pass filter (LPF) and band pass filtered (9-15) data;
2. the normalized time series of band pass filtered F10.7 and AEBI, with a lag of 2 years

**II. ON THE TIME SERIES NONLINEARITY**

1. The nonlinearity testing by correlation coefficients based on MI
2. Nonlinear links (Hsieh)
3. Linear/ Nonlinear test (Smith)

**A) The nonlinearity testing by correlation coefficients based on MI**

Vu *et al.* (2018)^1^ and Zaidan *et al*. (2018)^2^ demonstrated the advantage of the non-linear correlation coefficient bazat pe Mutual Information (MI) over the linear ones, such as Pearson and Spearman coefficients.

Nonlinear links between terrestrial variables were found in Mares *et al*. (2020)^3^ by comparing the linear to nonlinear correlation coefficient (NLR), calculated as follows:

$$NLR=\sqrt{[1-\exp(-2MI)]}$$

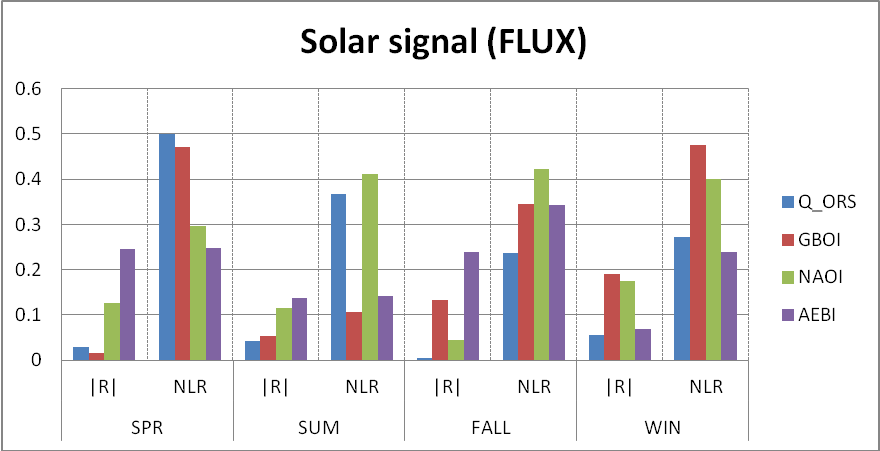


*Figure* S4. Linear (|R|) and nonlinear (NLR) correlation coefficients between solar flux and the four terrestrial variables for the simultaneous correlations in each of season in the 1948-2000 period.

In most cases, the values of nonlinear correlation coefficients are much higher than those of the linear correlation coefficient. Figure S2 shows a case in which the difference between linear and nonlinear correlations are large (solar flux with discharge at Orsova during spring)

a)

b)

*Figure S5*. a) Time variations in spring (1948-2000) of solar flux (red line) in comparison with the discharge at Orsova (blue line) b) scatter plot of the two time series. The both time series are standardized.

**B) Nonlinear links (Hsieh)**

Further, we will show another way to highlight the linear or nonlinear character of the connection between two time series.

By the neural network method, principal component analysis (PCA) or canonical correlation analysis (CCA) has become possible the establishment of nonlinear links between phenomena, through generalized nonlinear regression equations^4-6^ [(Hsieh (2001)^4^; Hsieh (2004)^5^; Wu et al.(2004)^6^]. Following Hsieh (2004)^5^ we can intuitively show the linear/nonlinear dependence of two variables.

As can be seen (Figs. S6, S7) even for relatively short time series, nonlinear connections can be highlighted both between geoclimatic time phenomena and between solar/geomagnetic activity and geoclimatic phenomena. (Fig. S9). But not for all the elements presented here we find links that deviate from linearity. So that, the nonlinear approach does not make sense for them.

*Figure S6*. Nonlinear canonical correlation analysis (NLCCA) mode 1 between NAO and Q-ORS in the winter time shown as a string of small circles in comparison with the dashed line which represents the linearity case.

*Figure S7.* Nonlinear canonical correlation analysis (NLCCA) mode 1 between GBOI and Q-ORS in the spring time shown as a string of small circles in comparison with the dashed line which represents the linearity case.

*Figure S8*. The evolution of the geomagnetic index (*aa*) and the NAO index in the winter season for the period 1901-2000. Both series are standardized.

*Figure S9.* Nonlinear canonical correlation analysis (NLCCA) mode 1 between *aa* (geomagnetic index) and NAO index in the winter time shown as a string of small circles in comparison with the dashed line which represents the linearity case.

1. **Linear/ Nonlinear test (Smith)**

A robust method to measure nonlinear dependence between two variables is described using mutual information, that analyses the linear and nonlinear components of dependence, separately^7^. This method has not yet been applied to the time series in the present study.

The overall proportion of the linear dependence to all dependence between *X* and Y will be measured by $\Lambda$, where

MI is mutual information of (X,Y), $MI'$ is mutual information of the inverse of cumulative distribution function of the residuals, from linear regression, if the inverse exist.

If MI > 0 , it indicates all dependence is completely nonlinear;

If $\Lambda$ = 1, the relationship is entirely linear and can be completely described

by the linear correlation coefficient.

**III. POSSIBLE MECHANISMS OF THE SOLAR/ GEOMAGNATIC SIGNATURE ON THE TERRESTRIAL VARIABLES**

1. ***QBO role in modulation of the influence of solar forcing***

As the climate system is known to be a dissipative, nonlinear system with many sources of instability, it is very difficult to provide a clear physical explanation of the impact of solar activity on climate variables, as from cause to effect. We hope that our results will help those who make complex models of the solar impact on the climate.

In the publication Mares et al. (2016)^8^ is analyzed Quasi-Biennial Oscillation (***QBO) role in modulation of the influence of solar forcing.***

To test this hypothesis, we selected the winter months in years with East QBO phase, and correlations between solar flux and terrestrial variables were calculated.

The correlation coefficient between the solar flux and the unfiltered winter European Blocking Index (EBI) for all those 53 years (1948-2000), is 0.15 and not statistically significant. By selecting only the years with QBO in the East phase in the winter months (34 cases), the correlation coefficient is 0.32 at the confidence level around 95%. It is interesting that although the power spectrum (Fig. 9a) highlights significant peaks related to the QBO (2.4 and 2.7 years), the correlation coefficient between EBI and QBO is insignificant. This suggests that the spectral representation is very useful in time series analysis and the QBO phases modulate the connection between solar activity and blocking circulation. These findings related to the QBO role are in accordance with the results obtained by Barriopedro et al. (2008)^9^, Huth et al., (2009)^10^, Sfîcă et al. (2015)^11^.

Cnossen and Lu (2011)^12^ presented some of the mechanisms which explain the QBO role in the solar signature in climate variables. These mechanisms have been supported by both observational and modeling studies, but some of them are yet unclear.

Composite maps^8^ enlighten the solar impact on atmospheric circulation in the lower troposphere, during the East phase of QBO, when the solar maximum is associated with blocking event over the northern Atlantic and north-western Europe (Fig. S10a), and the solar minimum to a geopotential with an opposite distribution (Fig. S10b). Sfîca et al. (2015)^11^ specify that through these composite maps nonlinearities are taken into account, at odds to using linear methods. Barriopedro et al. (2008)^9^ found similar results, namely QBO is a modulator of the transformation of atmospheric circulation from a blocking type circulation to a zonal one and vice versa, under the solar impact.

We mention that during 1948-2000, 34 winter months (DJF) were recorded in which East QBO phase occurred and the solar flux has produced atmospheric blocking events in the lower troposphere, or a zonal atmospheric circulation at middle and higher latitudes, depending on the state of maximum or minimum solar activity, respectively.


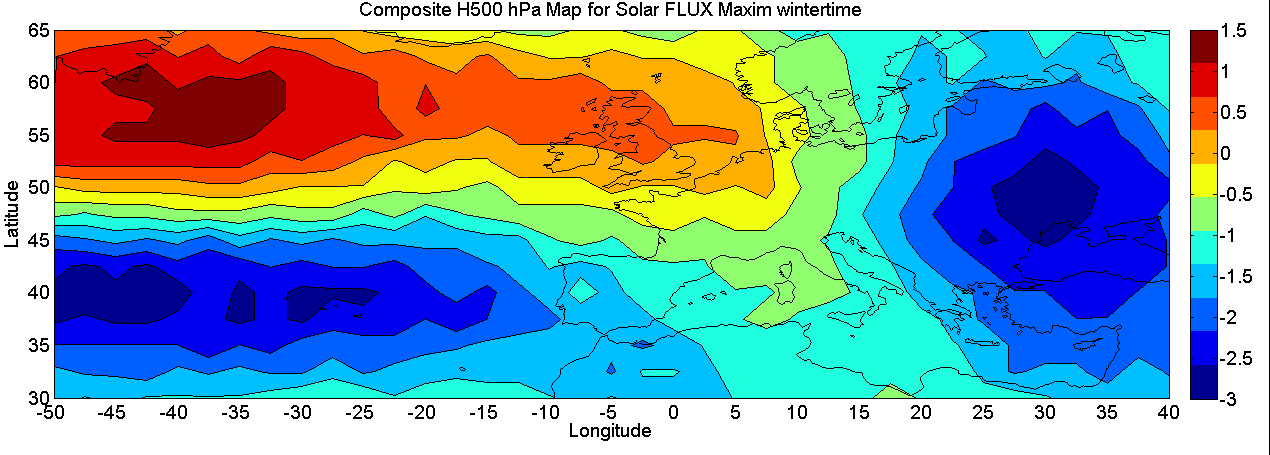


a)


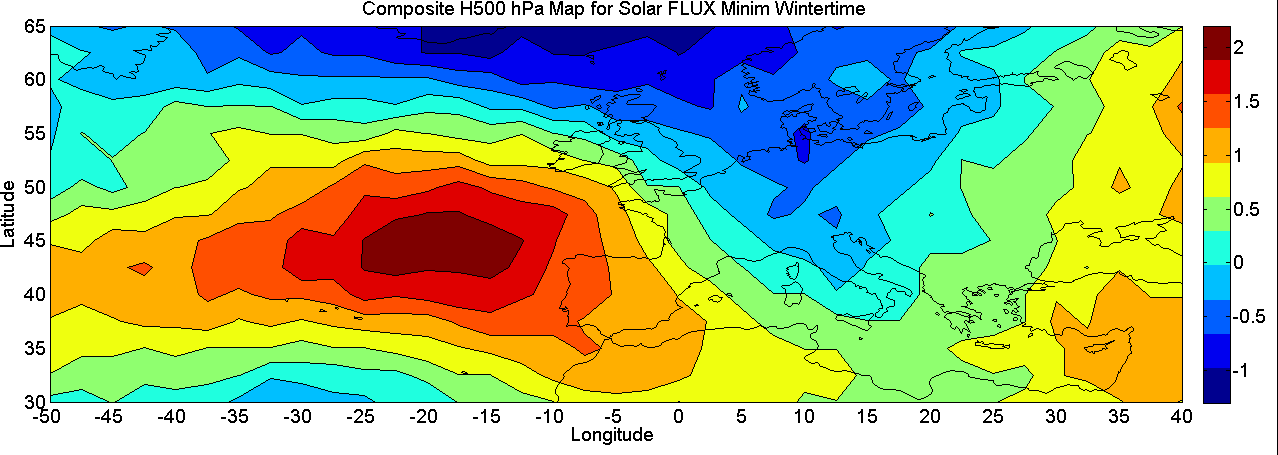


b)

***Figure S10.*** Composite maps for the winter H500 hPa anomalies, corresponding to solar flux associated with the east phase of QBO (1948-2000) and: a) maximum flux b) minimum flux

1. ***Why is our paper more rigorous than others?***

In the present study, the superiority of the chain of approaches in relation to the classical ones is justified by the assertion that the links between phenomena and mutual determinations are made by adapting the method to the specifics of natural nonlinear/linear evolution.

This means that approximating the nonlinear connections between phenomena, must not be coarse . So, let's see what type of connection there is, for example, between geomagnetic activity index (*aa*) and global mean temperature (GT). We considered the geomagnetic index *aa* and GT after reanalysis performed by Brohan et al. (2005) ^13^. Through classical linear analysis^14,15^, the authors find links in a first linear approximation.

Taking into consideration the classical method presend in El-Borie and Al-Thoyaib (2006)^14^ we can mention:

- The analysis is interesting in that the authors find significant linear correlations because they resort to segmenting data on portions with quasi-linear evolution;

- If the analysis had been done according to nonlinear techniques, it may have discovered other important characteristics in the connection of the geomagnetic index *aa* with global temperature at the level of the Earth's surface, which they analyzed;

- It can clearly see in Fig. S11, that the links of the two parameters *aa* and TG are nonlinear, following the neural network technique (Hsieh and Tang 1998) ^16^. In Fig. S11 it was noted with x1 represent the geomagnetic index, x2,x3 - represents the TG series multiplied by factor 10 and is obtained after the reanalysis carried out by Brohan et al. (2006)^8^.

- It can see it from Fig. S11a,b, as both in the planes (x1,x2) or (x1,x3) and also in the spaces (x1,x2,x3) in Fig. S11d, the links are curves (with red circles). In plane (x2,x3) from the Fig. S11c, i.e. in the TG plane by itself, the fit is made after a perfectly straight line marked with red circles that follow the straight line interrupted in black.

- It is meritorious the final conclusion from the work^14^ which by simple methods emphasizes that,“The sensitivity of TG to *aa* geomagnetic index is significant and may be real”.

Of course, variations in the geomagnetic activity follow the solar cycle, but in turn should alter the solar impact on the climate (Dergachev et al., 2012)^15^. This type of mechanism can also be extended to hydroclimatic variables like those analyzed by us in this paper, leading us not only to functional links between geophysical parameters through the use of mutual informational entropy but also the meaning of causal/reciprocal determination if it exists, by transfer entropy.

From what we know from previous our analyses, between terrestrial phenomena for example NAO and atmospheric blocking, there are interdependent so that in one period, one fenomenon is active, and determines on the other fenomenon (passive) and in another period the roles are reversed. It remains that in the following approach, we analyze in detail, the causal and the meaning of the connection between hydroclimatic variables by transfer of entropy. This analysis would lead us to the outline of truthful mechanisms in hydroclimatic processes.

***Figure S11***. The link between the global average annual temperature and geomagnetic activity *aa* from 1901-2000. x1-represents the geomagnetic *aa* and x2,x3 are the average global annual temperature multiplied by 10.

This result does not diminish the importance of the result obtained by El-Borie and Al-Thoyaib (2006)^14^, but the fact that detail is lost due to the closer nonlinear connections.

**References**

1. Vu, T. M., Mishra, A. K. & Konapala, G. Information Entropy Suggests Stronger Nonlinear Associations between Hydro-Meteorological Variables and ENSO. *Entropy* 20(1) **38,** (2018).
2. Zaidan, M. A. *et al*. Exploring non-linear associations between atmospheric new-particle formation and ambient variables: a mutual information approach. *Atmos. Chem. Phys.* **18,** 12699-12714 (2018).
3. Mares, I., Mares, C. Dobrica, V. & Demetrescu, C. Comparative study of statistical methods to identify a predictor for discharge at Orsova in the Lower Danube Basin. *Hydrol. Sci. J.* **65,**  371-386 (2020 ).
4. Hsieh, W.W. Nonlinear principal component analysis by neural networks. *Tellus* **53**A, 599-615 (2001).
5. Hsieh, W. W. Nonlinear multivariate and time series analysis by neural network methods. *Rev. Geophys*. **42**, RG1003,doi:10.1029/2002RG000112 (2004).
6. Wu, A. Hsieh, W. W. , Shabbar A. The Nonlinear Patterns of North American Winter Temperature and Precipitation Associated with ENSO. *J. Clim*.**18**, (2005).
7. Smith, R. A mutual information approach to calculating nonlinearity. *Stat.* **4**, 291-303 (2015).
8. Mares, I., Dobrica, V., Demetrescu, C. & Mares, C. Hydrological response in the Danube lower basin to some internal and external climate forcing factors. *Hydrol. Earth Syst. Sci. Discuss*.**1-24**, 2016-304. https://doi.org/10.5194/hess (2016).
9. Barriopedro, D., Garcıa-Herrera, R. & Huth, R. Solar modulation of Northern Hemisphere winter blocking. *J. Geophys. Res.* **113**, D14118, https://doi: 10.1029/2008JD009789 (2008).
10. Huth, R., Pokorná, L., Bochníček, J. & Hejda, P. Combined solar and QBO effects on the modes of low-frequency atmospheric variability in the Northern Hemisphere. *J. Atmos. Solar-Terrestrial Phys.* **71**, 1471-1483 (2009).
11. Sfîcă, L., Voiculescu, M., & Huth, R. The influence of solar activity on action centres of atmospheric circulation in North Atlantic. *Ann. Geophys*. **33**, 207-215, https://doi:10.5194/angeo-33-207-2015 (2015).
12. Cnossen, I., & Lu, H. The vertical connection of the quasi-biennial oscillation-modulated 11 year solar cycle signature in geopotential height and planetary waves during NorthernHemisphere early winter. *J. Geophys. Res.* **116**, D13101, https://doi:10.1029/2010JD015427 (2011).
13. Brohan, P., Kennedy, J. J., Harris, I.,Tett, S. F. B. & Jones, P. D. Uncertainty estimates in regional and global observed temperature changes: A new data set from 1850. J. Geophys. Res. 111, D12106, https://doi:10.1029/2005JD006548 (2006).
14. El-Borie, M. A. & Al-Thoyaib, S. S. Can we use the aa geomagnetic activity index to predict partially the variability in global mean emperatures?. *Int. J. of Phys. Sci.* **1**, 67-74 (2006).
15. Dergachev, V. A., Vasiliev, S. S., Raspopov, O. M., & Jungner, H. Impact of the geomagnetic field and solar radiation on climate change. *Geomag. and Aeronomy.* **52**, 959-976 (2012).
16. Hsieh, W. W. & Tang, B. Applying neural network models to prediction and data analysis in meteorology and oceanography. Bull. Amer. Meteor. Soc. 79, 1855-1870 (1998).
